# Real and virtual worlds alike: Adolescents' psychopathology is reflected in their videogame virtual behaviors

**DOI:** 10.1371/journal.pone.0181209

**Published:** 2017-07-14

**Authors:** Aviv Segev, Hila Gabay-Weschler, Yossi Naar, Hagai Maoz, Yuval Bloch

**Affiliations:** 1 Shalvata Mental Health Center, Hod Hasharon, Israel; 2 Sackler Faculty of Medicine, Tel Aviv University, Tel Aviv, Israel; 3 Cybereason Inc., Tel Aviv, Israel; Ariel University, ISRAEL

## Abstract

Current research refers to videogames as a constant variable. However, games today are designed to be highly interactive and versatile: two players may be using the same videogame, but as a result of different using patterns, the game will not necessarily encompass the same content and gameplay. The current study examined the possible relationship between psychopathology and in-game playing patterns. We hypothesized that adolescents would play videogames differently, in a manner that would reflect their particular psychopathologies. We examined 47 male adolescents from three diagnostic groups: those suffering from externalizing psychopathologies, internalizing psychopathologies and controls. We performed a high-resolution examination of their gameplay, using in-game quantitative statistics mechanisms of two fundamentally different games, a structured racing game and an unstructured adventure game. While there was no difference in the groups' using patterns of the structured game, there was a high variability between the groups' using patterns when they were using a non-structured game. These findings suggest that virtual behavior in unstructured games is reflective of adolescent-players psychopathology, and might shed light on an unexplored facet of videogames research. Possible implications are discussed.

## Introduction

Playing videogames has become a central leisure activity in the lives of adolescents and young adults in particular [1], and there has been much debate about the effects of this activity, and its possible connection to psychopathology [2]. However, conclusions have been hard to gather, and criticism has emerged regarding both the methodologies used and possible biases [3–5]. Most studies have focused on two major dimensions: screen-time (i.e., the time spent playing games, and addictive patterns) [6–8] and the content of the games (specifically violent content) [4,9]. While some studies have been able to link screen-time and violent content to psychopathology [10], the results of other studies have not borne out such a link and have instead offered alternative explanations for these findings [11].

Most of the research that has been conducted in this field views the videogame as a static unit, with a certain given content, much the way a television show is perceived. Indeed, many of the methodologies that have been used to study the patterns and effects of television watching have also been utilized in research on videogames [12], and many studies have examined their combined effect [6,13]. Until several years ago, when the individual's interaction with videogames was limited to action and response, and the plot and gameplay was basically linear, these methods made sense; they allowed investigators to assume that a specific amount of time spent playing the same game-title was a constant exposure.

In recent years, several types of games that expand the concept of interactive media have come into use. For example, open-world ("sand-box") games are very common game-mechanics, also used in the popular genre of Massive Multiplayer Online Role Playing Games (MMORPG). In these games, the player heavily influences the gameplay (the plot of the game as well as the player's behavior and experience during the game), in such a way that a specific action taken by the player at a certain point in the game will substantially affect future pathways, enabling new possibilities while eliminating others. Thus, it is possible that different players playing the "same" game are actually playing and experiencing a very different virtual environment.

To date, very few studies have aimed at examining in-game playing patterns [14], and even fewer have looked at the relationship between psychopathology and in-game playing patterns [15–17] beyond screen-time [18] and violent content [19]. When gamers with or without psychopathology were compared, the outcome measure was performance-based, similar to the functionality of cognitive testing [15–17,20].

In the current study, we hypothesized that adolescents would play videogames differently, in accordance with their particular psychopathologies. Furthermore, we hypothesized that the more flexible and responsive (unstructured) the game was, the more pronounced the reflection of the player's psychopathology would be on the gameplay. Thus, in this pilot study we focused on studying how three groups of adolescents—those suffering from externalizing psychopathologies, internalizing psychopathologies, and controls—differed in the ways they played a complex interactive game as opposed to a structured game.

## Materials and methods

### Participants

Participants were 47 male adolescents, between the ages of 13 and 18, who were required to have engaged in at least four hours of computer gaming per week for at least two years.

Females were not recruited to the study given implications in the literature of extensive cross-gender differences in patterns of playing and gaming. Gaming experience was set as an inclusion criterion to avoid variability stemming from major differences in gaming proficiency. Patients were excluded if they suffered from a major sensory disorder (blindness, deafness); had a psychotic or autistic spectrum disorder; had any motor or coordination disorder that might have interfered with their use of a keyboard, mouse, or controller; or were known to abuse drugs. The patients were recruited from a child and adolescent psychiatric outpatient clinic and were grouped into two dimensionally different categories: the first was characterized in the clinical setting mostly by behavioral difficulties, externalizing anger and aggression, sensation-seeking and risk-taking behavior (including attention-deficit hyperactivity disorder [ADHD], oppositional defiant disorder [ODD] and conduct disorders). The second category was characterized mostly by issues of avoidance, anhedonia and lack of energy, internalization of mental-pain, self-depreciation, (e.g., depression, dysthymia, anxiety). Congruent to the DSM5 clustering, we attempted to create two distinct groups that would aid in highlighting possible alterations in gameplay patterns. Further, In order to enable clearer distinctions in these patterns, subjects suffering from co-morbidities that combined the two categories were excluded.

The control group participants (i.e., individuals without any diagnosed psychopathology) were recruited from the community at large. In order to be included in the study, the participants with psychopathologies were evaluated by a senior child and adolescent psychiatrist to confirm their diagnoses and assure that there would be no conflicting co-morbidities. Adolescents recruited from the community were briefly interviewed by a psychiatrist; they also filled in the Strengths and Difficulties Questionnaires (SDQ), a well-validated screening tool of psychopathology dimensions, to ensure a lack of gross psychopathology.

Recruitment took place from September 2012 until February 2015. The study was approved by Shalvata Mental Health Center institutional review board (IRB), and all participants and their parents signed written informed consent forms.

### Tools

Demographic data and information regarding habits of computer use were collected. Adolescents were assessed using several well-validated and common psychological questionnaires. The Buss-Perry Aggression Questionnaire (AQ) [21] is a self-report questionnaire designed to quantify levels of aggressiveness. It consists 29 Likert-like scale items addressing physical aggression, verbal aggression, anger and hostility. Higher scores in the AQ indicate higher level of aggression. The Barratt Impulsiveness Scale (BIS) [22,23], is a 30-item self-report questionnaire designed to assess impulsivity as a trait of personality and behavior. It is considered to be a corner-stone in the study of impulsiveness [24]. BIS is also rated using a Likert-like scale, and higher scores indicates higher level of impulsiveness. The Spielberger State-Trait Anger Expression Inventory (STAXI) [25,26] is a 44-item self-report questionnaire using Likert-like scale, aimed to assess anger both as a transient state of emotions and as a temperament construct. In this scale, as well, higher anger levels are manifested by higher scores. In addition, to assess participants' level of proficiency in the games that were used in the current study, participants had to choose one out of seven sentences describing possible levels of familiarity with the game, ranging from "never heard of this game" to "I've finished this game several times".

Two computer games were used. The first, Need for Speed: Shift (NFS), is a highly-structured racing game in which the player repeatedly races against nine other cars played by the computer on different tracks and in different cars; the player is rated according to "aggressiveness" or "precision", based on his/her racetrack behavior (aggressive behavior such as colliding with another car, drifting out of the racetrack, blocking opponents' car, etc.). The second game, Grand Theft Auto (GTA) IV, is an open-world action-adventure game, in which the player embodies Niko Bellic, an eastern European gangster with a traumatic past who has just arrived in a crime-infested "Liberty-City", modeled on New-York City.

Data regarding game performance was collected via the internal in-game statistics mechanisms of the games, meant to provide the player with quantifiable and comparable performance assessments.

The two games were played in random order, with a 15–20 minute recess between games. Players played 30 minutes of NFS, having received no prior instruction other than help in setting-up the game. This period was followed by an additional 30 minutes during which they were asked to focus on the track behavior they had been weaker in the first session (i.e., aggressive racers were asked to drive more precisely, and vice-versa). As for GTA, in order to introduce the players to, or refresh them regarding, the game's rules, they began by playing for a period of 20 minutes, from the starting point of the game; they too received no prior instruction other than assistance in game setup. After that they played for an additional 40 minutes but this time they started at an advanced phase, at a point at which a wider range of capabilities were at the players' disposal. At the beginning of this second stage of GTA playing, the examiner said "If you'd like, you can head to the next mission at point D marked on the map", with no other directive instruction regarding goals or expectations.

As each game collects and records dozens of parameters, the parameters compared in the current study were chosen according to several criteria. Specifically, they were required to: 1) reflect a major mode of behavior in the game (i.e., the number of kills, rather than the method of kill, for instance by pistol or rifle); 2) be able to be collected objectively with a good measure of reliability (i.e., derived directly from the games' statistics and displayed in quantifiable measures); 3) be able to be used to describe modes of behavior even when the game was being played for a relatively short time rather than until the game's natural conclusion.

Therefore, the parameters examined in NFS were: 1) the amount, by percentage, of player aggression in the first and second 30-minute segments (i.e., participants played "naturally" in Part 1 and then "flipped" their track behavior in Part 2; 2) the change in players' aggressive behavior that resulted from the investigator's request to "flip" behavior; 3) the total number of races performed.

The parameters examined in GTA were 1) number of missions attempted, representing the player's efforts to follow the game's main storyline (missions were not measured according to the player's accomplishment of them, as such a measure would have been heavily reliant on game proficiency); 2) number of kills (i.e., acts of violence performed against game characters, and/or civilians who appeared in the game, by the player's avatar; it should be noted that these actions must be carried out at least at some level and are automatically rewarded according to the game's mechanics and contextual rules); 3) number of cars stolen (an unavoidable action in order to move around the game fictional "Liberty-City" but it can be performed repeatedly with no actual need and is not rewarded); 4) number of player deaths (events in which the game protagonist die, after which the game restarts at the beginning of the level); 5) number of police evasions, in percentages, a measure that encompasses two game measures: police run-ins (number and magnitude of conflicts with the police) and number of evasions (a necessary action in the game's inner context, as not evading the police would result either in the death of the character or in an arrest, which would negatively impact the character); 6) number of civilian run-downs—though possible and up to a certain point unavoidable, not a rewarded action in the game; 7) number of bullets fired; 8) shooting accuracy.

Our basic hypothesis was that behavior in the virtual world would reflect difficulties in the real world, when taking into account the context and rules of the virtual world. Thus, we assumed that function and achievements, when observing the game as a "mission" would be impaired in adolescents suffering from any diagnosis. Impaired scoring on measures that reflect attention, precision and impulsiveness would typify the externalizing group (e.g. adherence to games' goals, aggressive behavior, optimal use of available resources), while scoring that reflects avoidance, lack of energy and dysthymia would be impaired in the internalizing group (e.g., diminished activity in the game contextual world).

It should be mentioned that GTA is an M-rated videogame (i.e., intended for use above the age of 18) due to the violence exhibited in the game. However, studies show that 80% of children and adolescents play M-rated videogames, and 70% own such a game [27]. After careful deliberation with the IRB that approved this study, and considering the importance of using a common and familiar open-world game, we concluded that the possible harm from a one-hour exposure to GTA would be negligible. It should also be mentioned that as an inclusion criterion was at least four hours of computer gaming per week for at least two years, it was more than likely that the vast majority of the adolescents participating in the study had already been exposed to GTA; indeed, 83% of them reported being exposed to the game prior to the current study.

### Statistical analysis

Group demographics and performance were compared using an ANOVA for continuous variables; a chi-square test was used for categorical variables; and a Kruskal-Wallis test was used for ordinal parameters. We used an analysis of covariates (ANCOVA) to compare the game parameters between the three groups, while controlling for variables that might have confounded or skewed the results.

Due to the number of parameters examined in GTA, a principal component analysis containing all the GTA variables studied (with a loading factor of 0.6) was used to examine linked variables, and to reduce possible effects of multiple comparisons. The combined factors that resulted from the principal component analysis did not represent a tangible score in the game, but rather a mathematical function encompassing the clustered variables. These factors were compared between the three groups using ANCOVA, as described.

## Results

Of the 47 participants, 15 were controls, 12 were diagnosed with internalizing-cluster disorders, and 20 with externalizing-cluster disorders. The age ranges of the control, internalizing and externalizing groups were 14.4–17.5, 13.5–18.1, and 13.2–16.5 years, respectively. The mean age differed significantly between the groups, with a younger age range typifying the externalizing group. No significant differences were reported in computer screen-time between the groups.

An assessment of the groups revealed a significant increase of impulsiveness and aggression scores in the externalizing group and a borderline-significant increase of anger score (Table 1).

**Table 1:** Basic characteristics compared between the different groups.

	Controls (n = 15) mean (SD)	Internalizing (n = 12) mean (SD)	Externalizing (n = 20) mean (SD)	p Value
Demographics				
Age (years) ^a^	15.6 (0.93)	15.9 (1.7)	14.6 (0.91)	0.005
Housing Situation ^b^				n.s
Living with both parents	61.5%	58.3%	61.1%	
Living with one parent	30.8%	33.3%	27.8%	
Other	7.7%	8.3%	11.1%	
Socio-economic density ^a^ (rooms per people at home)^a^	1.28 (0.38)	1.51 (0.67)	1.50 (1.27)	n.s
Average daily computer screen-time (hours)	4.18 (2.52)	4.92 (2.69)	4.41 (3.11)	n.s
Psychological Assessments				
Aggression Questionnaire ^a^	63.87 (18.30)	67.83 (11.57)	81.58 (20.60)	0.016
Barratt Impulsiveness Scale ^a^	58.93 (11.97)	61.50 (5.90)	67.18 (8.56)	0.038
Trait Anger ^a^	7.40 (4.61)	9.17 (5.17)	11.05 (3.75)	0.068
Game familiarity				
NFS ^c^				0.096
No familiarity	46.7%	83.0%	60.0%	
Some familiarity	26.7%	16.7%	35.0%	
Extensive familiarity	26.7%	0.0%	5.0%	
GTA ^c^				n.s
No familiarity	20.0%	58.3%	25.0%	
Some familiarity	40.0%	8.3%	50.0%	
Extensive familiarity	40.0%	33.3%	25.0%	

^a^ Examined using ANOVA

^b^ Examined using chi-square

^c^ Examined using Kruskal-Wallis

There were borderline-significant differences in familiarity with the NFS game, with the internalizing group being less acquainted with it. No significant differences were observed in GTA game familiarity. Group characteristics are presented in Table 1.

### NFS results

NFS parameters were compared between the groups using a univariate analysis of covariance (ANCOVA). We entered age, aggression score, impulsiveness score and game familiarity, as they differed between the three groups, as covariates (a borderline difference was observed in game familiarity between the three groups, and this difference was assumed not to be related to the difference associated with the psychopathology group). No significant differences were found in any of the parameters, meaning in the level of aggression presented in both parts of the game, in the ratio of behavioral change after the investigator's intervention, and in the number of total races played during the two sessions (Table 2).

**Table 2:** Univariate analysis of NFS parameters.

Parameter	Controls (n = 14) Mean (SD)	Internalizing (n = 12) Mean (SD)	Externalizing (n = 20) Mean (SD)	p Value ^a^	Adjusted r^2^
Aggression ratio—Part 1	0.56 (0.06)	0.55 (0.08)	0.54 (0.07)	>0.1	n.a
Aggression ratio—Part 2	0.52 (0.05)	0.54 (0.06)	0.54 (0.06)	>0.1	n.a
Aggression change upon request	0.11 (0.05)	0.12 (0.12)	0.09 (0.12)	>0.1	n.a
Number of total races played	14.08 (1.50)	16.25 (2.56)	14.88 (3.28)	>0.1	n.a

^a^ Adjusted for age, aggression score, impulsiveness score and game familiarity, as a covariate

### GTA results

A principal component analysis yielded a grouping of the eight variables measured into three mutually exclusive weighted factors. Examining the grouping of variables from the in-game contextual perspective, one group contained three variables that were measures of goal-directedness and game progression (missions attempted, evasion of police, and player deaths). An additional group contained three variables that formed the core of activity in the game (killing, firing and shooting accuracy), which were naturally linked together and were organically rewarded as a result of how the game is designed. The last group was composed of two variables, neither of which were rewarded actions and neither of which contributed to the game progression; although both of these actions represented redundant wandering, they had to be executed at some level in the GTA virtual world (stealing cars and running down civilians).

As with the NFS parameters, GTA factors were analyzed using ANCOVA, entering age, aggression score and impulsiveness score as covariates (Table 3).

**Table 3:** Univariate analysis of GTA parameters.

Parameter	Controls (n = 14) Mean (SD)	Internalizing (n = 11) Mean (SD)	Externalizing (n = 19) Mean (SD)	p Value ^a^	Adj. r^2^
Progression Factor **	0.68 (0.84)	-0.28 (1.12) ^b^	-0.34 (0.84) ^b^	0.011	0.24
Missions attempted *	6.57 (2.28)	3.58 (2.31) ^b^	4.05 (2.78) ^c^	0.009	
Ratio of police evasions	0.57 (0.28)	0.36 (0.29) ^b^	0.36 (0.22) ^c^	0.068	
Number of player deaths	3.42 (2.77)	5.18 (3.15) ^c^	5.84 (2.48) ^b^	0.078	
Activity Factor	0.31 (0.63)	-0.43 (0.70) ^c^	0.14 (1.20)	>0.1	n.a
Number of kills	24.57 (9.65)	16.73 (11.22)	24.26 (17.28)	>0.1	
Number of bullets fired *^+^	505.57 (204.76)	312.92 (168.12) ^b^	455.11 (295.81) ^c^	0.069	
Shooting accuracy	0.60 (0.09)	0.49 (0.10) ^b^	0.51 (0.13)	0.054	
Wandering Factor	-0.36 (1.06)	-0.26 (0.77)	0.38 (0.96) ^b^	0.056	n.a
Number of cars stolen	12.86 (7.57)	12.67 (5.26)	17.16 (6.60) ^b^	0.057	
Number of "run-downs"	19.86 (10.74)	23.00 (13.04)	32.63 (18.28) ^b^	>0.1	

^a^ Adjusted for age, aggression score and impulsiveness score as covariates

^b^ Significant difference from controls in contrast analysis

^c^ Borderline-significant difference from controls in contrast analysis

* Age was borderline-significant as a covariate

** Age was significant as a covariate

^+^ Aggression score was borderline-significant as a covariate

The Progression factor showed significant differences between the three groups. A contrast analysis of ANCOVA (with the control group as the reference group) was performed to compare each group to the control group, revealing that it was the control group which differed from the other two groups. A post-hoc analysis of the three variables comprising the Progression factor revealed that the control group had significantly more mission-attempts compared to the internalizing group (p = 0.002) and a borderline-significant more attempts compared to externalizing groups p = 0.096). In addition, the control group's tendency to evade a detrimental police encounter was borderline-significant, and a contrast analysis comparing each of the groups yielded a significant difference between the control and internalizing groups (p = 0.029) and a borderline-significant difference between the control and externalizing groups (p = 0.085). The tendency of the control group to "die" at a lower rate was borderline-significant (p = 0.078), and a contrast analysis showed a significant decreased "dying" of the control group compares to the externalizing group (p = 0.038) and a borderline-significant similar decrease compared to the internalizing group (p = 0.083).

No significant difference was observed in the Activity factor between the three groups. However, a contrast analysis yielded a borderline-significant difference between the internalizing group and the control group (p = 0.087), implying decreased activity of the internalizing group. A post-hoc analysis of the variables comprising the Activity factor revealed no difference in the number of kills between the three groups or any difference in the contrast analysis (p>0.1 for all). The number of bullets fired borderline-significantly differed when comparing the three groups, and a contrast analysis comparing each group to the control group showed a significant decrease in shots fired of the internalizing group compared to the control group (p = 0.032) and a borderline-significant decrease of the externalizing group compared to the control group (p = 0.073). An ANCOVA revealed a borderline-significant difference in shooting accuracy (p = 0.054), and a contrast analysis showed a significant decrease in accuracy between the control and the internalizing groups (p = 0.018) and a non-significant difference between the control and externalizing groups.

The Wandering factor (comprising actions that are not rewarded by the game mechanics) yielded a borderline-significant difference. A contrast analysis, with the control group as the reference group, revealed a significant difference between the externalizing group and the control group (p = 0.021), and a non-significant difference between the internalizing group and the control group (p>0.1). When analyzing the two variables comprising the Wandering factor, a borderline-significant difference was observed in the number of cars stolen (p = 0.057). A contrast analysis yielded a significant difference between the externalizing group and the control group (p = 0.031), and a non-significant difference between the internalizing group and the control group (p>0.1). The number of "run-downs" of bystanders did not significantly differ between the groups, though a contrast analysis showed a significant increase in the externalizing group compared to the control group (p = 0.034), and no significant difference between the internalizing group and the control group (p>0.1).

## Discussion

The central finding of this pilot study is that player attributes have a significant effect on the non-structured videogame being played. The study also showed that this variability of behavior does not exist in relation to structured games, a finding which is in keeping with the existing literature [15,16,20].

The analysis of the Progression factor showed that both internalizing and externalizing subjects adhered significantly less to the game's contextual missions than did the controls. They also seemed to "die" more often and evade the police less often, thus enduring additional negative consequences. These behaviors are probably in accordance with in-game risk-taking behavior in general. These results align well with commonly held views that psychopathology-free children are able to successfully advance in day-to-day tasks (scholastic, social, emotional, etc.) and with fewer detours and complications than are children with psychopathologies. Results regarding the Wandering factor seem, as well, to follow the same logic; i.e., the externalizing group behavior was found to be more aimless and "destructive" than the internalizing or control group behavior, with no in-game justifications.

No difference was found in the Activity factor between the three groups, a somewhat surprising finding given the fact that children with externalizing psychopathologies might be expected to fire and kill more, especially in light of the Wandering factor, while children with internalizing psychopathologies might be expected to sit the game out. Several explanations can be offered regarding the externalizing group. First, when looking at the results overall, while the level of activity was the same, among the externalizing group this activity was directed in ways that were not to their benefit. An additional explanation might be that the aimless roaming and police run-ins that typified the psychopathology groups might have resulted in a depletion of ammo and weapons, leading to apparently similar numbers of kills and shootings. Another important point to keep in mind, even when considering the positive results of the other two factors, is that the group heterogeneity (i.e., ADHD, ADD, ODD and conduct disorders) created an artificial mean that does not truly represent each group. For example, it might be that ADHD children who have a more hyperactive symptomatology differ from those whose salient symptomatology is inattentiveness. However, due to the pilot nature of the study, we lacked the statistical power to examine such a hypothesis. As for the internalizing group, a borderline significant-difference was observed in the activity factor, which might suggest that the differences are too minute to be observed in our pilot study. The fact that the progress of this group was impaired, with no compensating "wandering" compared to the control group, raises the question of what they were doing during the game. These borderline results might contain a clue to the answer.

Thus, it is not only that games can be played differently by different people, but also that the gameplay differences are aligned with basic features of the diagnostic group. In other words, while there was no statistical difference in the activity level between the groups and with high variability regardless of psychopathology, participants utilized the activity differently. Specifically, healthy (control group) subjects made significantly more gains in the game than did the other two groups. Moreover, the externalizing group, characterized primarily by impulsive, risk-taking and aggressive behavior (mostly ADHD and ODD), harnessed this activity to unrewarded actions, perhaps mirroring the way their distractibility and impulsivity work to their detriment in "real life." Conversely, the internalizing group did not, as the control group did, engage in the plotline of the virtual world, nor did it engage in the mayhem of the externalizing group. A trend towards lower activity over-all is therefore suggested—aligning with the avoidant, dysthymic, and energy-lacking traits that typify this kind of group. This novel finding suggests that the same mental and psychological traits that challenge children in the real world may challenge them in the virtual gaming world as well.

It is important to mention that our study did not detect differences between the groups in terms of the amount of time they spent, on average, in front of the computer: a finding which comes in contrast to previous studies conducted among this age group [6,28]. A possible explanation for this finding is the selection bias of relatively heavy users—gamers, who ipso facto use their computers more intensively.

The findings from the current study have several possible implications.

First, they may have a substantial bearing on research done in the field of videogames.

Although most of the scientific literature has not differentiated between structured and "open" games, such a differentiation might aid in clarifying some of the conflicting findings that have been published [15–17]. For example, to date, research that has focused on game-performance by groups suffering from psychopathologies has focused mainly on highly structured games (which can be monitored, analyzed and compared more easily) [15–17], and this focus is perhaps the reason why no differences have been reported, except in patterns of use (e.g., gaming time) [19].

Going forward, we might question the meaning of a "videogame" as a substrate of any study. Two players may report using the same game, but the actual playing process may be experienced very differently by them. GTA, used in our study, serves as a good example: A necessary run-in with the police can end in a quick getaway. Alternatively, depending on the action taken by the player, it can evolve into a multi-casualty, helicopter-aided manhunt enhanced by spectacular graphics of urban landscape destruction. Although many studies have explored "the game," what the game actually *is* has often remained unclear: is it meant to be an endless spree of destruction, or is it intended to be a focused journey to achieve certain goals?

It would be interesting to further analyze whether the different kinds of gameplay (and thus, game experience) affect players differently. When it comes to studying the effect of television shows, it is only natural to assume that when the same show is presented to different subjects, the exposure is basically the same. Up until about ten years ago, such an assumption would have been equally logical in the field of videogames, due to their relatively "closed" or limited plotlines. However, as many of the newer videogames are highly adaptable to players' actions and choices, one must ask—when it comes to open-world videogames—whether the exposure can be viewed in such a monolithic way.

As different players are likely to create different gameplay, they might—in turn—be exposed differentially [29]. Thus, when studying the effects of open-world videogame exposure, a systematic bias might exist. While this question cannot be answered on the basis of the current study, the results lend support to the importance of investigating the "player" variable when studying videogames, in addition to studying the variables that have already been pointed out in the literature, such as content, context, structure, mechanics and time [2].

A second possible implication of the study's findings relates to the use of gameplay as an assessment tool for psychopathology. In this study, it has been shown that the diagnostic group is influential regardless of age and psychological attributes of aggression, impulsivity and anger. As the field of psychiatry on the whole is eager to develop tests that will capture the essence of a disorder, it is possible that the wide range of behaviors that are manifested in virtual environments encapsulate many of these hidden dimensions within a quantitative monitored environment. Psychological tests, cognitive as well as others, are heavily affected by motivation, interest, anxiety, setting and other factors related to the subject, the tool, and the context [30–32]. Thus, despite efforts to measure a specific attribute of the subject as precisely as possible, the artificial nature of the testing procedure itself colors the results (the observer effect) [33]. Using videogames, however, might lessen such obstacles: the question of motivation and even more so, of interest, would no longer be as relevant, and the point of the test would remain hidden from the player, thus diminishing factors of cooperation and malingering. In the current study, the differences that arose between the diagnostic groups were not reducible to core psychological elements of aggression, impulsivity and anger. This finding suggests that the game may offer a sort of gestalt that captures multiple aspects of behaviors and represents the core psychopathology better than unidimensional measures can.

While questionnaires and unidimensional tasks are aimed at a content-based outcome, the use of videogames allowed us to examine what the subject's approach towards the questionnaire was and how he used it. In the case of GTA, what we discovered was that the subjects, though they used the same amount of firepower, chose to fire for different reasons, a finding that would not have emerged if the question hadn't been viewed within the gameplay context. This example is analogue to two children who answer fewer questions on an exam than their classmates do. A tester measuring only the number of correct answers would not be able to account for the "why" of these results; however, a gameplay-analysis as proposed by our study would reveal that one of the children was more hesitant to answer questions, while the other child used half his/her exam time transforming the questionnaire form into a paper airplane.

An additional finding of our study bears further examination. Structured games would seem to focus on relatively uniaxial capabilities of the player—such as speed, accuracy, and visuospatial abilities. The fact that in those games we found no difference between children with psychopathology and healthy controls (nor did previous studies [15,16]), is puzzling, as these games often utilize and measure attributes similar to the ones that cognitive tests utilize and measure to evaluate ADHD. While our study cannot provide a definitive answer to this question, a possible explanation would be that immersion in the game can sift oppositional attitudes, or just plain lack-of-interest, which might be more prominent in the classical, less attractive, computerized cognitive tests.

The current study had several limitations.

First, given the pilot nature of our study, our sample was small. Second, the setting of the study was not the "natural" setting in which videogames would ordinarily be played; rather they were played in a laboratory located within a mental health center. While this setting was similar to that in which assessment tools are commonly applied, it might have altered the playing behavior of the children in a differential manner. Moreover, they did not play a game of their choosing, a factor which might also altered motivation and, consequently, playing behavior [14]. Third, the children played the games only once, and therefore our results cannot attest to whether the differences reflected a trait or state variation. Fourth, the context of the unstructured game used in this study was of a criminal-nature; making progress in the game therefore meant being successful in enacting criminal behavior, at least virtual criminal-behavior. This element raises important questions regarding the measurement of health behavior in such a virtual world. It is possible that a "true" control group would be made up of children who would rather not play GTA, though it would then be very complicated to make a comparison among the groups. Finally, several parts of the games required an acquaintance with the English language, and the level of the children's proficiency in English was not measured. However, most of the children had played GTA prior to the study and were familiar with the game and its rules.

It should be noted that while this study examined different behavioral patterns within a given mode of a game, many videogames nowadays allow multiple playable modes. The player can choose (or be attracted to) only one of them, or several combined. Examples of such modes include games that offer a melee (e.g., fighting skill tournaments) vs. a quest (fighting with-in a context and towards a moral goal). They also include single player modes (in which the player is focused on a story) vs. multiplayer modes (which include extensive social elements, such as team planning, altruism and clan commitment). These aspects further enhance the richness of information that might be embedded within a gameplay process regarding the player him/herself [34].

## Conclusions

This study revealed that psychopathology was associated with adolescents' different in-game playing patterns. Moreover, these differences were shown to be aligned with basic features of the adolescents' psychopathologies. Examining these differences might expand the field of research into videogames in a way that will contribute to the understanding of the intricate associations between videogames and psychopathology. Moreover, if the current findings are verified by additional larger studies, an examination of the differences in playing patterns of children with psychopathologies might lead to the development of a novel assessment tool for diagnosis, evaluation and follow-up.

## Supporting information

S1 File Data set. The data-set underlying the findings of the current study, SPSS Format.
(SAV)
